# Optimal management of cervical cancer in HIV-positive patients: a systematic review

**DOI:** 10.1002/cam4.485

**Published:** 2015-07-01

**Authors:** Atara Ntekim, Oladapo Campbell, Dietrich Rothenbacher

**Affiliations:** 1 College of Medicine, University of IbadanIbadan, Nigeria; 2 Institute of Epidemiology and Medical Biometry, Ulm UniversityUlm, Germany

**Keywords:** Cervical cancer, HIV-positive, management

## Abstract

The clinical management of cervical cancer in HIV-positive patients has challenges mainly due to the concerns on immune status. At present, their mode of management is similar to HIV-seronegative patients involving the use of chemotherapy and radiotherapy concurrently as indicated. HIV infection, cancer, radiotherapy, and chemotherapy lower immunity through reduction in CD4 cell counts. At present there are no treatment guidelines for HIV-positive patients. This study was done to systematically review the literature on cervical cancer management in HIV-positive patients and treatment outcomes. A systematic literature search was done in the major databases to identify studies on the management of HIV-positive patients with cervical cancer. Identified studies were assessed for eligibility and inclusion in the review following the guidelines of The Cochrane Handbook for Systematic Reviews and CRD's (Centre for Reviews and Dissemination) guidance for undertaking reviews in health care. Eight eligible studies were identified from the literature. Three of them were prospective while five were retrospective studies. Notably, the average age at diagnosis of cervical cancer in HIV-positive patients was a decade lower than in seronegative patients. There was no difference in distribution of stages of disease at presentation between HIV-positive and negative patients. Mild acute toxicity (Grades 1 and 2) was higher in HIV-positive patients than in HIV-negative patients in hematopoietic system. In the grades 3 and 4 reactions, anemia was reported in 4% versus 2% while gastrointestinal reactions were reported in 5% versus 2% respectively. In general, patients who were started early on HAART had higher rates of treatment completion. The study supports the suggestion that HAART should be commenced early at cervical cancer diagnosis in HIV-positive patients diagnosed with cervical cancer to ensure less toxicity and better treatment compliance.

## Introduction

Cancer of the uterine cervix is the most common gynecological malignancy and occurs worldwide 1. It has been reported that about eighty percent of cervical cancers occur in the developing countries 2. Furthermore, the mortality due to cervical cancer is higher in the developing countries where screening and treatment modalities are not commonly available or accessible compared with the developed countries 3. According to the GLOBOCAN 2012 estimates, the highest incidence is in sub-Saharan Africa, especially in Eastern African countries 4. The most important risk factor for the development of cervical cancer is the human papilloma virus (HPV) 5.

Cervical cancer is very common in HIV-seropositive patients with an aggressive course and poor treatment outcome 6. Regions of high prevalence of cervical cancer correspond with regions of high prevalence of HIV infection 7. Cervico-vaginal HPV infection has also been reported to be higher in HIV-positive women than in their HIV-negative counterparts. In a study involving 1778 HIV-positive and 500 HIV-negative women, it was found that 63% of the HIV-positive participants tested positive to HPV viral DNA while only 30% of the HIV-negative participants tested positive 8. HIV-positive women have been reported to have seven times more incidence of cervical cancer than their HIV-negative counterparts 9.

HIV infection lowers immunity through the destruction of CD4 lymphocytes. The level of destruction is related to the patient's HIV viral load 10. Progressive reduction in CD4 cell population reduces the ability of the body to ward off infective agents leading to occurrence of opportunistic infections in HIV infected individuals. Dormant infections can also be reactivated under conditions of suppressed immunity. These opportunistic infections add to the deterioration of the clinical states of HIV infected patients leading to poor outcome. Opportunistic infections are common if CD4 cell count is below 200 cells/*μ*L 11.

The three modes of treating cervical cancer are surgery, radiotherapy, and chemotherapy either single or in combination. In areas with high prevalence of cervical cancer, majority of the patients are treated with chemo-radiotherapy which can lower patients' immunity 12,13. Highly active antiretroviral treatment (HAART) has helped to improve the immunological status of HIV-positive patients and control the increase in viral load 14. Patients with compromised immunity usually suffer more treatment toxicities from chemo-radiotherapy used in the treatment of cervical cancer as it affects the immune status of patients 15. The recovery of CD4 cell depends on the state of the thymus gland as they are thymus dependent. The thymus gland undergoes involution in adults and hence recovery of CD4 cell count is usually very slow in those with involute thymus gland. In a study to assess the activity of the thymus gland after chemotherapy, it was reported that in younger patients aged between 18–49 years, the thymus function was evident in 63% of the participants compared with 0% of their counterparts aged 70–91 years 3 months post treatment 16.

Another consideration in HIV-positive patients with cancer is that some chemotherapy and HAART drugs are metabolized by similar cytochrome p450 enzyme pathway. This may affect the clearance of chemotherapy drugs leading to increased toxicity or ineffectiveness 17.

Various treatment modalities and modifications have been used to improve outcome of treatment in HIV-positive patients with cervical cancer. However, the outcome of management is still poor. There is a need to explore ways of improving the effectiveness of treatment and limit potential-associated increased toxicity in these patients. Presently, optimal uniform treatment modalities are yet to be established.

The objectives of this study were to systematically summarize the current available data on mode of treatment and outcome of treatment in HIV-positive patients and compare to HIV-negative ones and to further identify existing gaps in studies regarding the management of patients with cervical cancer and HIV.

## Methodology

The method used followed the guidelines of The Cochrane Handbook for Systematic Reviews Version 5.1.0 (http://handbook.cochrane.org/; accessed 17 February 2015) and CRD's (Centre for Reviews and Dissemination) guidance for undertaking reviews in health care (http://www.york.ac.uk/inst/crd/pdf/Systematic_Reviews.pdf; accessed 17 February 2015). The report is presented according to the recommendations of Preferred Reporting Items for Systematic Reviews and Meta-analysis (PRISMA) 18. Ethical review of the protocol was not required as this is a systematic review of already published (and therefore anonymized) data.

### Sources of Data

A systematic search in electronic data bases of the scientific literature was used. The data bases searched included MEDLINE (PubMed interface), EMBASE (OVID interface), Cochrane Central (OVID interface), Web of Science, Cochrane data base, The NHS Centre for Reviews and Dissemination, Centre for Evidence Based Medicine at Oxford, Scopus, Google Scholar, Chinese BioMed (CBM) and online published doctoral theses from January 1995 until March 2014. The period from 1995 was chosen because HAART was widely introduced into the management of HIV infection at this point in time 19,20. Relevant materials were also obtained through communication with known experts. Clinical Trial.gov database was also searched to identify completed trials that were relevant to the study.

### Search Strategy

A systematic search strategy with structured terms of medical subject headings (MeSH) and free keywords were used. The following words were used for this search: ‘treatment of HIV patients with cervical cancer’, ‘cervical cancer in HIV patients’, ‘chemo-radiation in HIV patients with cervical cancer’ and ‘HIV with cervical cancer treatment side effects’. There was no restriction on the study language although the search was only done in English language. Four full articles written in other languages were translated into English for evaluation using google translator. The review was carried out by two people (AN and OC). The study abstracts were first identified from the databases and other sources. They were checked to remove duplicates. Then screening was done according to our eligibility criteria to remove irrelevant studies. Full texts of the screened studies were obtained for further evaluation for eligibility. In addition, retrieved articles were also checked whether any further related articles could be found (cross-references). Eligible studies were selected for the systematic review.

### Eligibility Criteria

Studies were included if they consisted of a sample size of more than 20 HIV-positive subjects and met the following criteria.

### Population of Interest

Included studies were those with target populations with primary conditions of interest which are patients with cervical cancer and HIV seropositivity. In the case of studies involving HIV-positive and HIV-negative patients with cervical cancer, the results of the HIV subgroup were extracted for the analysis if other study criteria were met. Studies focusing on any of the main objectives namely survival, toxicity, and treatment tolerability were also evaluated.

### Interventions of Interest

Studies involving the use of radiotherapy/chemotherapy with or without Highly Active Antiretroviral Treatment (HAART) were considered for the review. Concurrent chemo-radiotherapy is considered a standard of care for cervical cancer and the addition of HAART depends mainly on the CD4 cell count of the patient.

### Comparators

The outcome of treatment in HIV-positive patients with cervical cancer was compared with the outcome in cervical cancer patients who were HIV-seronegative and received similar interventions.

### Study Outcomes

The primary outcome of interest in this study was survival. This included local control, progression-free survival and loco-regional and distant failure. Secondary endpoints included acute and late toxicity, treatment compliance or adherence and CD4 cell count trend of patients during treatment.

### Study Designs

Observational studies, randomized controlled trials (RCTs), and controlled clinical trials (CCTs) for interventions were included. Quasi experimental studies that contained most of the information required were also included. The publication types included were full peer reviewed original papers and published doctoral thesis (if not published otherwise). Reviews, case series, and case reports were excluded since they may not provide needed details for evaluation or do not fulfil our minimal sample size requirements.

### Data Extraction

The data extracted included study identification, number of patients and age range, diseases characteristics, treatment modalities, toxicities, treatment compliance, and outcome among others. The data were extracted using a self-designed data extraction form.

### Assessment of Methodological Quality of Studies

The quality of the studies was assessed based on whether the information of interest relative to meeting the aims of this study were included. In addition, the quality of interest that the studies to be included in the review should have included information about HIV-positive patients with cervical cancer treated with radiotherapy (external beam + brachytherapy), cisplatin chemotherapy and HAART as indicated. In addition, the studies should also include information on acute and late reactions during treatment assessed in major systems namely skin, hematological (hemoglobin, white blood cells and platelets), neurological, gastrointestinal system, and genito-urinary system using standard toxicity scales. The response rates based on the FIGO stages and treatment compliance were to be assessed as well and all these compared with HIV-negative patients.

### Assessment of Quality of the Studies for Risk of Bias

The study quality was assessed according to the guidelines on presentation of assessments of risk of bias as outlined in Cochrane Handbook for Systematic Reviews for Randomized Trials 21. The GRACE (Good ReseArch for Comparative Effectiveness) principle was also employed to assess the quality of observational studies (www.graceprinciples.org; accessed 17 February 2015). The latter is a validated checklist for evaluating the quality of observational cohort studies for decision-making support.

These various domains of the studies were classified into low risk, high risk, and unclear risk (of bias) based on the assessment using the following main domains:

1. Were the study plans (including research questions, main comparisons, outcomes, etc.) specified before conducting the study?
2. Was the study conducted and analyzed in a manner consistent with good practice and reported in sufficient detail for evaluation and replication?
3. How valid is the interpretation of comparative effectiveness (CE) for the population of interest, assuming sound methods and appropriate follow-up?

The check list used for study quality assessment is presented in Table1. In the table, different sets of criteria are used to assess prospective and observational studies because observational studies although do provide valuable information, are relatively weaker in methodology compared to RCT and hence a consensus principle is used to guide the assessment of observational studies 22. The determination of the risk of bias was done by means of personal assessment since the use of scoring quality scales or checklist is discouraged by Cochraine Reviews (http://handbook.cochrane.org/chapter_8/8_3_3_quality_scales_and_Cochrane_reviews.htm; accessed 17 February 2015). Studies were therefore assessed as having ‘low risk’ of bias if the study appears to be free of sources of bias. ‘High risk’ of bias was described if there was at least one important risk of bias for example, the study had a potential source of bias related to the specific study design used or had some other identified issue. Unclear risk of bias was ascribed in cases where there may be a risk of bias, but there is either: insufficient information to assess whether an important risk of bias exists; or insufficient rationale or evidence that an identified problem will introduce bias. A more comprehensive list of criteria for assessing quality of the studies is contained in Table S1.

**Table 1:** HIV and Cervical cancer treatment outcome: quality assessment check list for the studies

*Quality assessment domains-Prospective trials RCT/non RCT*		
Participation bias	Yes	No
Population of interest is adequately described for key characteristics		
Study setting and geographic location is adequately described		
Baseline sample is adequately described for key characteristics		
Inclusion and exclusion criteria are adequately described		
Patients were balanced in all aspects except the intervention		
Attrition bias	Yes	No
Follow-up is sufficiently long for outcome to occur (≥6 months)		
Proportion of sample completing the study is adequate (≥80%)		
Description of withdrawal (incomplete outcome data) is provided		
Characteristics of drop-outs versus completers is provided		
Outcome measurement		
Definition of outcome is provided a priori		
Objective definition of outcome is provided		
Data analysis and reporting	Yes	No
Alpha error and/or beta error is specified a priori		
Data analysis was based on intention-to-treat analysis principle		
Frequencies of most important data (for example, outcomes) are presented		
*Quality assessment domains – Retrospective studies*		
Data	Yes	No
Were treatment and/or important details of treatment exposure adequately recorded for the study purpose in the data source?		
Were the primary outcomes adequately recorded for the study purpose (e.g., available in sufficient detail through data source		
Was the primary clinical outcome(s) measured objectively rather than subject to clinical judgment (e.g., opinion about whether the patient's condition has improved		
Were primary outcomes validated, adjudicated, or otherwise known to be valid in a similar population?		
Was the primary outcome(s) measured or identified in an equivalent manner between the treatment/intervention group and the comparison group(s)?		
Were important covariates that may be known confounders or effect modifiers available and recorded?		
Methods	Yes	No
Was the study (or analysis) population restricted to new initiators of treatment or those starting a new course of treatment?		
If one or more comparison groups were used, were they concurrent comparators? If not, did the authors justify the use of historical comparisons group(s)?		
Were important covariates, confounding and effect modifying variables taken into account in the design and/or analysis?		
Is the classification of exposed and unexposed person-time free of “immortal time bias”?		
Were any meaningful analyses conducted to test key assumptions on which primary results are based?		

RCT, randomized controlled trial.

### Data Analysis and Presentation

The data extracted from the studies are summarized and presented in tables and figures. Descriptive analyses including proportions/percentages are also used to describe the data. A Summary of Findings (SoF) table is used to summarize the main findings from the studies.

## Results

### Literature search

**Figure 1:**
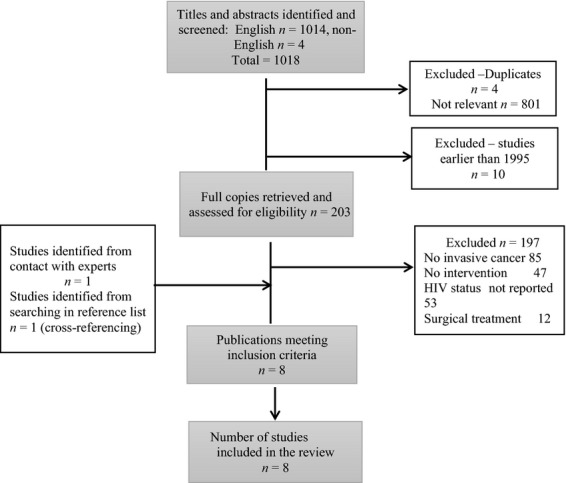
Flow diagram showing the literature search process with associated data.

A flow diagram showing the process of the literature search with associated data is displayed in Figure1. Initial search identified 1018 potentially relevant citations including four citations in non-English language literature (I French, 1 Italian, I Spanish and 1 Portuguese).

Four duplicate entries were removed including 801 studies that were not relevant. Ten studies conducted before 1995 were also excluded following initial screening of the titles and abstracts. Further assessment of full texts of the remaining 203 publications led to the exclusion of 197 studies. The reasons for their exclusion are depicted in Figure1. Eight studies met the criteria for inclusion in the studies. There was one RCT, two prospective non-randomized studies, and five retrospective studies. Search on ClinicalTrials.gov (performed May 5, 2014) did not identify any completed study relevant to this topic.

### Assessment of methodological quality of the studies

The quality assessment level of the included studies based on GRACE principle and Systematic Reviews principles for retrospective and randomized control trials respectively (Table1) are presented in Table2. In summary, the overall assessment of the included studies was low especially in terms of quality of treatment assessment, treatment allocations, patient's selection, and follow-up.

**Table 2:** Results of the methodological quality assessment of studies on HIV patients with cervical cancer. Studies with low levels of bias in at least two domains were assessed as of high overall quality (details see methods section)

Study type (reference)	Risk of bias		
	Study methods	Data analysis	Overall assessment
Retrospective studies			
Gichangi et al. 43	High	High	Low
Moodley and Mould 44	High	High	Low
Shrivastava et al. 45	High	Uncertain	Low
Simonds et al. 29	Low	Low	High
Al-Noseery 27	High	Uncertain	Low

	Participation	Attrition	Data analysis	Overall assessment
Prospective studies				
Gichangi et al. 15	High	High	High	Low
Msadabwe 26	High	Low	Uncertain	Low
Munkukpa 23	High	Low	Low	High

### Study characteristics

We did not find any RCT study that randomized HIV-positive patients with cervical cancer on one arm and HIV-negative patients with cervical cancer on the other arm with HIV-positive patients receiving external beam radiotherapy, brachytherapy, chemotherapy with HAART (if indicated based on CD4 cell count). Such studies were also expected to assess the toxicity and response pattern among the two groups. The characteristics of the studies included in the review are summarized in Table3.

**Table 3:** HIV-positive patients with cervical cancer: main characteristics of the studies included in the review

Study, reference	Sample size	Study period	Country	Study type	Characteristics of cervical cancer									Median age (years)
					Stage (%)						Histology (%)			
					I	IIA	IIB	IIIA	IIIB	IVA	Squamous	Adeno	Others	
Gichangi 43	36	1989–1998	Kenya	Retro	NA	NA	NA	NA	NA	NA	NA	NA	NA	NA
Moodley 44	45	2003	South Africa	Retro	I-IIA (14%)		IIB/IIIA (27%)		IIIB/IVA (49%)		89	4	7	41
Shrivastava 45	42	1997–2003	India	Retro	5	7	29	–	45	5	98	2	–	41
Gichangi 15	41	2000–2002	Kenya	Prosp (NR)	2.4	–	63.4	–	31.7	2.4	87	5.3	7.7	38.1
Msadabwe 26	25	2009	South Africa	Prosp (RCT)	NA	NA	NA	NA	NA	NA	NA	NA	NS	39.5
Simonds et al. 29	59	2007–2010	South Africa	Retro	I/II (16.9%)			III/IV (83.1%)			94.9	1.7	3.4	41
Munkukpa 23	55	2012	Zambia	Prosp (NR)	13	7	60	2	18	–	98.2	1.8	–	40
Al-Noseery 27	137	2012	Zambia	Retro	NA	NA	NA	NA	NA	NA	NA	NA	NS	42

Sq, squamous cell carcinoma; Adeno, adenocarcinoma; Retro, retrospective cohort study; Prosp, prospective; NR, nonrandomized; RCT, randomized controlled trial; NA, not available.

The description of the characteristics of patients varied among the different studies. Five studies reported the average age of HIV-positive patients with cervical cancer while only three reported such among HIV-seronegative patients. Similarly, reports on histology and stage of malignancy also varied with no uniformity in the reports. The stages are grouped under early (FIGO stages I to IIB) and late (FIGO stages IIB to IVA) as depicted and summarized in Table4.

**Table 4:** Patients characteristics – HIV-positive versus HIV-negative cervical cancer patients from the various studies

	No. of studies that reported item	HIV-positive (%)	No. of studies that reported item	HIV-negative (%)
Median age (years) (Range years)	5	40 years (27–70)	3	52 years (30–80)
Histology	3	Sum of the 3 studies (N/T)	2	Sum of the 2 studies (N/T)
Squamous		130/142 (92)		448/491 (91)
Adenocarcinoma		3/142 (2)		25/491 (5)
Others		9/142 (6)		18/491 (4)
Stage	3		2	
I-IIA (early)		15/138 (11)		44/491 (9)
IIB-IVA (late)		123/138 (89)		447/491 (91)

*N* = sum of the item within a parameter (histology, stage), T = Total of all items within a parameter.

### Toxicity assessment

The pattern of reports on the toxicity and treatment compliance was also very variable among the included studies. Some reports graded toxicity under grades 1 and 2, then 3 and 4 while others separated the various grades. Other studies did not give any information on the toxicity and the proportion of patients that complied with the treatment. The results are summarized in Table5.

**Table 5:** Pattern of toxicity and treatment compliance reported by the studies on HIV-positive patients with cervical cancer

			Toxicity (%)	
Study	Treatment	Toxicity scale	GIT	GUS	Hem	Skin	Treatment completionon schedule (%)
Gichangi et al. 43	EBRT NS	G1	NS	NS	NS	NS	NS	NS
	Brachy NS	G2	NS	NS	NS	NS	NS	
	CTH NS	G3	NS	NS	NS	NS	NS	
	HAART NS	G4	NS	NS	NS	NS	NS	
Moodley and Mould 44	EBRT Yes (dose NS)	G1	NS	NS	NS	NS	NS	53%
	Brachy NS	G2	NS	NS	NS	NS	NS	
	CTH NS	G3	NS	NS	NS	NS	NS	
	HAART NS	G4	NS	NS	NS	NS	NS	
Shrivastava et al. 45	EBRT (45–50 Gy)	G1	45	46	NS	NS	RTOG	52%
	Brachy (25–30 Gy –54% of patients)	G2	41	18	NS	NS		
	CTH-Nil	G3	9	–	NS	NS		
	HAART NS	G4	5	–	NS	NS		
Gichangi et al. 15	EBRT (40–50 Gy)	G1	NS	NS	NS	NS	NS	
	Brachy Nil	G2	NS	NS	NS	NS		
	CTH Nil	G3	} 34	20	–	39		67%
	HAART Nil	G4	NS	NS	NS	NS		
Msadabwe 26	EBRT 45 Gy (Mean)	G1	5	8	15	NS	RTOG/WHO	72%
	Brachy 24 Gy in 3#	G2	7	1	7	NS		
	CTH Cisp 30 mg/m^2^ weekly	G3	2	–	4	NS		
	HAART Yes	G4	–	–	–	NS		
Simonds et al. 29	EBRT 46–50 Gy	G1	NS	NS	NS	NS	NS	45%
	Brachy 20–26 Gy	G2	NS	NS	NS	NS		
	CTH Cisp 40 mg/m^2^	G3	NS	NS	NS	NS		
	HAART Yes	G4	NS	NS	NS	NS		
Munkukpa 23	EBRT 46–50 Gy	G1	63	58	34	49	NCI-CTC	75%
	Brachy 20-26 Gy	G2	33	35	17	47		
	CTH Cisp 80 mg/m^2^ 3 weekly	G3	–	–	5	5.5		
	HAART Yes	G4	–	–	5	–		
Al-Noseery 27	EBRT 46–50 Gy	G1	84	79.6	84	NS	NCI-CTC	77%
	Brachy 26 Gy	G2						
	CTH Cisp 80 mg/m^2^ 3 weekly	G3	6.8	6.1	12.2	NS		
	HAART Yes	G4						

EBRT, external beam radiotherapy; Brachy, brachytherapy; CTH, chemotherapy; NS, not stated; GUS, genitourinary system; GIT, gastro intestinal system; Hem, hematopoietic system; G, grade, #, fractions of brachytherapy; RTOG, Radiation Therapy Oncology Group; NCI-CTC, National Institute-Common Toxicity Criteria.

There was only one study that assessed the acute toxicity in HIV-positive compared with HIV-negative patients treated with radiotherapy, chemotherapy, and HAART 23. In the study, response rate was not reported and late toxicity was also not assessed. This report is summarized in Table6.

**Table 6:** Toxicity in HIV-positive versus HIV-negative group-single report (*N* = 55) (from 23

Site of toxicity (organ system)	HIV-positive		HIV-negative control		*P*-value
	Treatment (events/patients)	%	Treatment (events/patients)	%	
Acute Gd 1/2					
Anemia	14/55	25	20/55	36	0.2
White cell count	38/55	69	28/55	51	0.06
Platelets	21/55	38	9/55	16	0.019
Genitourinary	52/55	95	53/55	96	0.709
Gastrointestinal	54/55	98	54/55	98	1
Neurological	0/55	0	0/55	0	1
Dermatological	53/55	96	55/55	100	0.5
Acute Gd3/4					
Haemoglobin	2/55	4	1/55	2	not stated
White cell count	5/55	9	5/55	9	
Platelets	0/55	0	0/55	0	
Genitourinary	0/55	0	0/55	0	
Gastrointestinal	3/55	5	1/55	2	
Neurological	0/55	0	0/55	0	
Dermatological	1/55	2	0/55	0	

### CD4 cells count

The CD4 count records were not consistently reported. Some studies used CD4 count of 200 as the minimum level to commence HAART which was the earlier WHO recommendation 24 while only one study among those that reported CD4 count level used CD4 count of ≤350 as minimum level to commence HAART which is the current WHO recommendation for ordinary HIV-positive patients 25.

One study 26 reported the CD4 cells count trend during the treatment and up to 3 months after treatment period. The average initial CD4 cells count was 321.06 cells/mm^2^ at commencement of treatment. This gradually dropped to 62.56 cells/mm^2^ at the end of treatment giving a mean difference of 258.5 cells/mm^2^. There was however, gradual rise after treatment by 3 months which was the end of the follow-up period of the study, but the pretreatment level was not reached. The average count at the end of 3 months was one-third of the pretreatment value.

### Summary of findings

The acute toxicity profiles following treatment with complete modalities were available in three studies 23,26,27. They are presented as Grades 1 and 2 and Grades 3 and 4 in Table7. Since there was only one study that reported toxicity in a comparative group (HIV-positive vs. HIV-negative), historical data from studies on HIV-negative group were used to put the numbers in context. The data were from a meta-analysis by Cochrane reviews 28).This work assessed the toxicity of chemo-radiotherapy in cervical cancer patients who were HIV negative.

**Table 7:** Summary of findings: study participants (HIV-positive treated with cisplatin and radiotherapy from REFERENCES) versus historical cancer controls (HIV-negative and treated with cisplatin and radiotherapy)

Toxicity	Cancer patients (HIV (positive) from references			Historical cancer controlsChemoradiotherapy for Cervical Cancer Meta-analysis Collaboration 28) (HIV-negative)		
	Number of trials	Treatment (events/patients)	%	Number of trials	Treatment (events/patients)	%
Acute Grades 1/2						
Site of toxicity						
Hemoglobin	3	110/217	51	4	373/700	53
White cell count	3	160/217	74	8	667/1326	50
Platelets	3	35/217	16	7	273/1296	21
Genitourinary	3	155/217	71	7	228/946	24
Gastrointestinal	3	188/217	87	10	585/1150	51
Neurological	3	0/217	0	4	64/740	9
Dermatological	3	170/217	78	6	180/1011	18
Grade 3/4 Toxicity						
Site of toxicity						
Hemoglobin	3	16/233	7	4	25/429	6
White cell count	3	17/233	8	11	265/1479	18
Platelets	3	5/233	2	8	26/1356	2
Genitourinary	3	8/233	4	6	9/1106	0.8
Gastrointestinal	3	33/233	9	13	156/1516	10
Neurological	1	0/31	0	5	7/867	1
Dermatological	3	23/233	10	8	28/1329	2
Response						
Complete	2	38/72	53	10	589/1349	44
Partial	2	34/72	47	10	220/1374	16

Information on treatment compliance was not consistently reported in the studies that met the study modalities of treatment. Some patients skipped chemotherapy and continued with radiotherapy due to renal compromise after some doses of cisplatin. Some skipped due to toxicity while others skipped some treatments due to disease progression (malignancy or HIV or both) and development of opportunistic infections which were not primarily linked with treatment toxicity.

The response rate presented were the ones reported by only two studies – (Msadabwe 26 and Simonds 29) whose patients had complete modalities of treatment. The methods of assessing and concluding on complete or partial responds (PAP smear or radiological methods) were not stated in any of the studies.

They are summarized as complete and partial response. These are also compared with data extracted from historical studies reported in Cochrane reviews and are included in Table7.

## Discussion

This systematic review summarized available evidence regarding the treatment of cervical cancer patients with HIV infection treated with chemo-radiotherapy which is a mode of treating cervical cancer recommended by many treatment guidelines. Unfortunately, there was no published RCT comparing the treatment outcome in HIV-positive patient with HIV-negative counterparts. The average quality of the included studies was rather low and most of observational nature with suboptimal control for various forms of bias and confounding factors. This reveals a gap in evidence-based management of HIV-positive patients diagnosed with cervical cancer.

All the studies included in the report were carried out in developing countries. This supports the fact that both, cervical cancer and simultaneous HIV burden of disease are high in these regions. This is also reflected in the management modes for these patients. Up to 2005, some centers were not able to treat their patients fully with external beam radiotherapy, brachytherapy, chemotherapy, and HAART. This was probably due to inadequate facilities. An analysis by International Atomic Energy Agency on radiotherapy facilities in 52 African countries noted that, although cancer incidence is increasing in the region only 23 countries had teletherapy facilities while only 20 had brachytherapy treatment units. This situation (which still prevails) cannot guarantee effective treatment of cancer cases 30.

This review shows that median age of HIV-positive cervical cancer patients was at least a decade lower than in their HIV-negative counterparts (40 years vs. 52 years). This has been attributed to the fact that the high virulence and hence progression of HPV infection to cause invasive cervical cancer is faster in HIV-positive patients than their seronegative ones 31. In terms of histological types, the predominant histological cell type is squamous cell carcinoma in both HIV-positive and HIV-negative patients.

The result of this study also shows that there is no major difference in the proportion of patients with early disease (stages I-IIA) from late disease stages IIB-IVA between HIV-positive and HIV-negative (early disease 11% vs. 9%, late disease 89% vs. 91%). This finding is different from the report by Maiman et al. 32 stating that patients who tested HIV positive usually have more advanced cervical cancer than HIV-negative patients 32. This may be because in areas with high incidence of cervical cancer which corresponds with low resource settings, access to screening and early treatment of the disease is low and most patients with cervical cancer present late, irrespective of HIV status 33–36.

The toxicity assessment varied considerably in the included studies and the assessment methods were not uniform. Most are a mixture of toxicities from different chemotherapy and radiotherapy doses in addition to various stages of cervical cancer and HIV. Looking at the proportion of patients that completed the prescribed treatments, those on HAART have higher rates of treatment completion than those without. This confirms earlier findings that patients on HAART are more likely to tolerate chemo-radiotherapy than those without 37.

Still, the coverage of HAART treatment in patients with HIV in some countries is not adequate. A report from Cameroon stated that about 58% coverage was achieved and this was stated to be an improvement. There are logistic issues and lack in facilities, funds and human resources to implement the programs of HIV management in low resource countries 20,38,39. CD4 cells count assays were also lacking in some studies possibly due to unavailability.

The single study that reported toxicity profiles among HIV-positive and negative patients showed that acute toxicity grades 1 and 2 were significantly higher in HIV-positive patients (for platelets 38% vs. 16% *P* = 0.019). In the acute grades 3 and 4 reactions, Hb was 4% versus 2% while GIT reactions were 5% versus 2%, respectively. These reactions were manageable and 75% of the patients completed their treatment. The reactions in other systems were essentially similar. In that report, all the HIV-positive patients were placed on HAART irrespective of CD4 cell count status according to the institutions' protocol. In the other study by Al-Noseery 27 including patients from the same center, the percentage treatment completion rate was 77%. This observation points to the direction that the commencement of all HIV-positive patients with cervical cancer on HAART irrespective of the CD4 cells count status can ensure a better treatment compliance and hence a better outcome. This is also supported by the fact that in the WHO clinical staging for HIV infections, cervical cancer occurrence in an HIV-positive patient classifies the patient as stage 4 alongside with other AIDS-defining malignancies like Kaposi's sarcoma and lymphoma. In these patients classified as stage 4 infection, it is strongly recommended that HAART should be commenced with CD4 cell count of ≤500 cells/mm^3^ 25.

Furthermore, the trend in CD4 cells count reduction reported in one of the studies 26 is quite informative. An average reduction in CD4 cell count of 258.2 cells/mm^2^ during treatment shows that patients with normal CD4 cell count can experience very low cell counts during therapy. The recovery of CD4 was also slow as it was noticed that at 3 months post therapy, average CD4 count was one-third of initial level. In addition to this point justifying the commencement of HAART, patients' CD4 count should be monitored closely to know when to commence them on prophylaxes for opportunistic infections like pneumocystis carinii and tuberculosis.

HIV-positive patients on chemotherapy are also known to have increased viral load if they are not on HAART. The viral load decreases as soon as HAART is introduced 40.

The toxicity profile presented in table7 compares acute toxicity from the studies with historical data from systematic reviews and meta-analyses on HIV-negative studies from the literature. Although the sample size difference between the two groups is quite wide and though it may be a comparison with many limitations, certain observations can still be made. In the mild acute reaction groups (grades 1 and 2) reactions are more on the HIV-positive group than HIV-negative in all the systems—WBC, GUS, GIT, and skin. These grades of reactions do not usually lead to treatment interruptions. In the moderate–severe acute reactions (grades 3 and 4) the differences were more in genitourinary and dermatological reactions. The analysis of response rates included in the two studies that reported these differences was not done based on stage of the disease and whether HAART was received or not. The report is presented considering all the patients irrespective of the stage of the disease and whether oncology treatment was completed or not.

An important result in the report by Simonds et al. 29 was that disease stage and completion of radiotherapy (at least 68 Gy) were the only independent factors that predicted good disease control 29. Similarly, the completion of at least three courses of chemotherapy has been reported to ensure benefit from chemotherapy 41.

Factors that usually lead to poor adherence to treatment schedule include toxicity and poor performance status. The use of HAART has been shown to minimize these in HIV-positive patients 23.

Simmonds et al. 29 also suggested that chemotherapy could be withdrawn from HIV-positive patients with stage IIIB and above disease to enable them complete radiotherapy with less toxicity 29. We think that if such patients are on HAART, their tolerance to chemo-radiotherapy will be better and the additional benefit of adding chemotherapy is desirable. This is based on the 2008 report of The Meta-Analysis Group, Medical Research Council Clinical Trials Unit, London, United Kingdom 42 on a systematic reviews and meta-analysis on using radiotherapy and concurrent chemo-radiotherapy in the management of cervical cancer. Fifteen trials were analyzed and it was concluded that there was an absolute benefit of 6% with chemo-radiotherapy over radiotherapy alone in disease-free survival and 8% benefit in disease control over 5 years. The effect was, however, found to be greater in early disease than with advanced disease. The absolute survival benefits were 10% (Stage IA to IIA), 7% (Stage IIB), and 3% (Stage III to IVA) at 5 years. Chemotherapy should, however, be withdrawn in cases with potential renal function impairment.

The level of disease control as reported in Table7 is quite remarkably compared with historical controls though the sample size was comparatively low. It suggests that there are some benefits in treating these patients with chemo-radiotherapy. In HIV-seronegative patients, response rate is highly dependent on stage of the disease with early disease performing better than late disease.

Fortunately, the adverse effects due to drug–drug interaction between cisplatin/carboplatin and most antiretroviral drugs that can be used in the treatment of patients with cervical cancer is mild though some may need minor dose adjustments as illustrated in The University of Liverpool HIV drug interactions website (www.hiv-druginteractions.org; accessed 21 October 2014).

## Limitations of the Study

The number of studies identified addressing the management of HIV-positive patients with cervical cancer were very few and the quality of the studies quite low. The mode of reports was also haphazard making it difficult to characterize the studies properly. In addition, outcome measures were difficult to be compared due to nonuniformity of study parameters and data. With regard to patients on HAART, the possible side effects associated with HAART which may influence the side effects of treatment in HIV-positive patients were not taken into consideration.

## Conclusion

Currently there is no standard guideline for the management of HIV-positive patients diagnosed with cervical cancer. These patients are managed like their HIV-seronegative counterparts. This is justified based on the results of this review but additional measures should be applied to HIV-positive patients such as commencing all patients on HAART since few studies that commenced all HIV-positive patients with cervical cancer on HAART reported better rates of treatment compliance comparable with their HIV-negative counterparts and with manageable toxicity. The completion of treatment which is an important factor for good disease control is also enhanced by HAART.

## Supporting Information

Table S1. Determining low and high risk of bias.
